# Capillary Hemangioma in Joubert Syndrome: A Case Report

**DOI:** 10.7759/cureus.38922

**Published:** 2023-05-12

**Authors:** Hala M Nassim, Reem A Alabdulqader, Hatim Najmi, Hend M Alsawadi, Hammam A Alotaibi

**Affiliations:** 1 Department of Ophthalmology, Imam Abdulrahman Bin Faisal University, Khobar, SAU; 2 Department of Ophthalmology, Dhahran Eye Specialist Hospital, Dhahran, SAU; 3 Research Center, Prince Sultan Military Medical City, Riyadh, SAU

**Keywords:** oral propranolol orbital hemangioma, pediatric genetics, molar tooth sign (mts), capillary hemangioma, joubert syndrome (js)

## Abstract

A baby girl who underwent cesarean section delivery and had a complicated postnatal course requiring neonatal intensive care unit (NICU) is followed in the pediatrics clinic for several months. At five months old, the baby girl was referred to an ophthalmology clinic with brain stem and cerebellum malformation consistent with the molar tooth sign (MTS) on magnetic resonance imaging (MRI) of the brain, hypotonia, and developmental delay. She has the classic features of Joubert Syndrome (JS). Other findings not typically associated with the clinical picture of the syndrome were observed in this patient, specifically skin capillary hemangioma of the forehead. Cutaneous capillary hemangioma was an incidental finding in this JS patient and responded favorably to medical treatment with propranolol where a significant reduction in the size of the mass was observed. This incidental finding can be seen as a potential addition to the spectrum of associated findings in JS.

## Introduction

Joubert syndrome (JS) is a rare genetic condition inherited in an autosomal recessive pattern with a prevalence of 1/100,000 live births. It was first described in a family of four siblings by Marie Joubert in 1969 [1]. Since then, the study of the disease has gained more attention, with a specific focus on the genetic component and the spectrum of systemic associations and presentations.

Patients will have unique findings that assist in the clinical diagnosis. First, a hypotonic state at birth that develops into ataxia as the infant grows. Second, an abnormal ocular movement (nystagmus). Finally, characteristic breathing difficulties with episodes of apnea and tachypnea. On physical examination, patients usually have occipital encephalocele, polydactyly, renal disease, hepatic fibrosis, and, to a lesser extent, involvement of other organs and systems. Moreover, ocular structures are affected by the disease, where the involvement of the retina leads to a dramatically low visual potential coupled with a possible failure of the normal development and function of the visual system, which precipitates the nystagmus commonly observed in such patients [2].

Patients with JS will also have a defining imaging characteristic that is considered pathognomonic when observed on magnetic resonance imaging (MRI), the molar tooth sign (MTS). This sign points to a malformation of the midbrain and hindbrain, typically occurring during prenatal development.

Infantile hemangiomas are classified as hamartomas and are considered the most common benign tumors among children [3]. Clinically, they are characterized by a red or blue raised lesion that appears in the third to seventh weeks of life. They typically continue to grow up until about five months of life, after which they tend to regress in size over a few years [4]. Infantile hemangiomas can be isolated or as part of other syndromes that are associated with vascular tumors, such as the PHACE syndrome (posterior fossa abnormalities, hemangiomas, arterial/aortic anomalies, cardiac anomalies, eye abnormalities), or syndromes associated with vascular malformations, including Sturge-Weber syndrome [5].

We report herein the case of a patient diagnosed with JS who developed a capillary hemangioma that was treated successfully with beta-blockers.

## Case presentation

**Figure 1:**
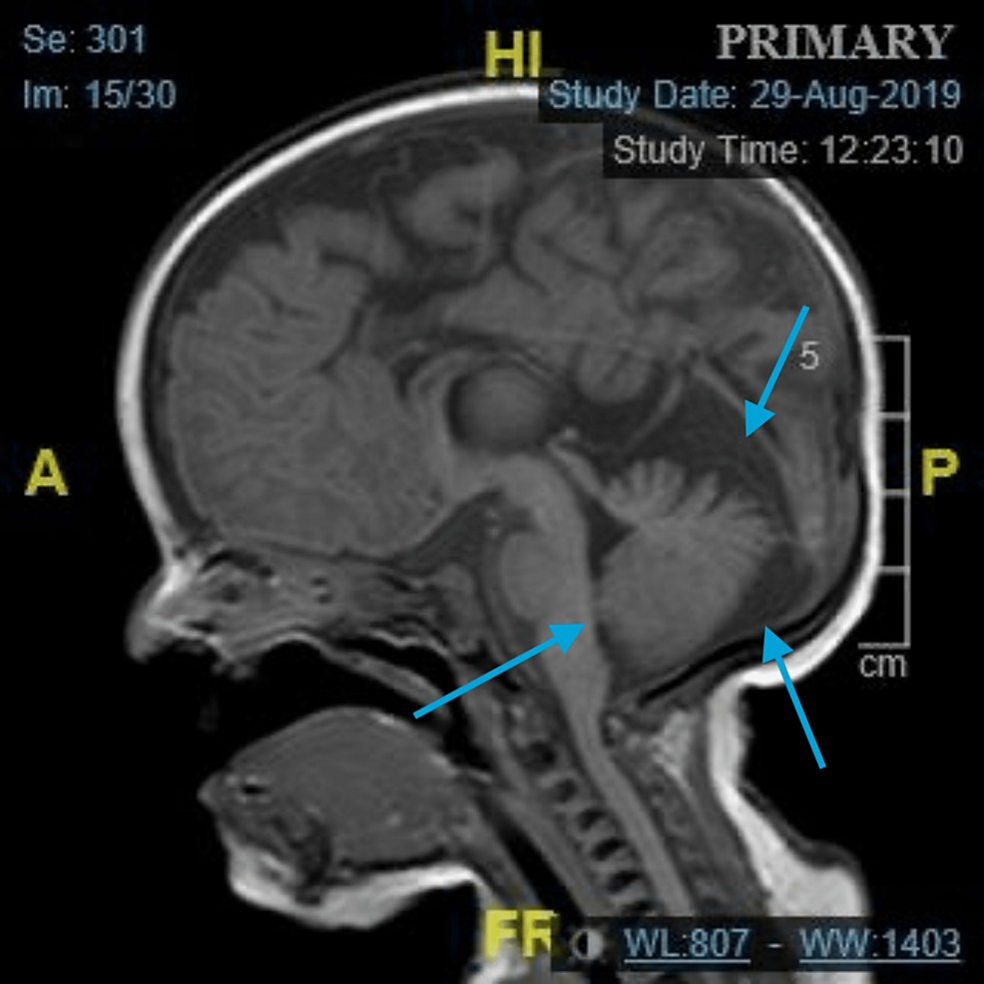
Sagittal section of T1-weighted MRI with vermian hypoplasia, shifting of the fastigium, and deep interpeduncular fossa (blue arrows). MRI: magnetic resonance imaging, A: anterior, P: posterior, Hl: head length, FR: frequency, CM: centimeter

**Figure 2:**
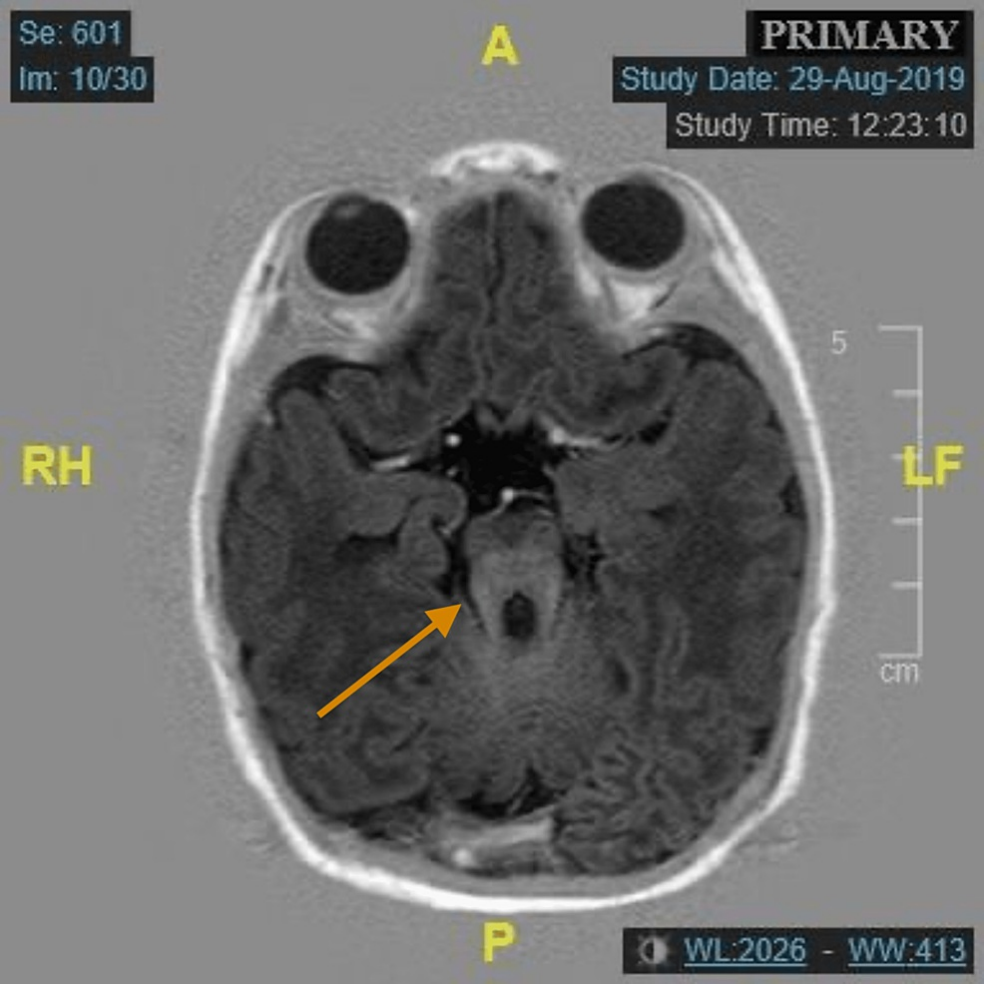
Axial section of a T1-weighted MRI showing the pathognomonic molar tooth sign (orange arrow). MRI: magnetic resonance imaging, A: anterior, P: posterior, RH: right, LF: left, CM: centimeter

This is the case of a baby girl who was delivered by cesarean section and admitted to the neonatal intensive care unit (NICU) due to multiple issues, including facial dysmorphic features, low birth weight, and breathing difficulties requiring positive pressure ventilation. The NICU course lasted more than 10 days during which she was stabilized and then discharged home. The baby underwent several examinations and investigations during the NICU stay and subsequent follow-up appointments in the pediatrics clinic. Given her dysmorphic features, MRI was done to investigate the extent of the anomalous changes in bone and soft tissue, where multiple features highly suggestive of JS were delineated through the imaging protocol. The MRI showed the characteristic MTS along with vermian hypoplasia/dysplasia, rostral shifting of the fastigium, and a deep interpeduncular fossa (Figure 1 and Figure 2).

At the age of five months, the patient was referred to the ophthalmology clinic and diagnosed by pediatrics as a case of JS. The patient was regularly followed in the pediatrics clinic; however, ophthalmology involvement was sought for evaluation and management of a newly developed forehead mass. A general inspection of the patient shows a young girl with dysmorphic features, hydrocephalus, hypotonia, developmental delay, bilateral cleft lip, and partial cleft palate. On her ocular examination, visual acuity testing shows eccentric, unsteady, and unmaintained fixation in both eyes. The patient failed to initiate a saccade, indicating the presence of ocular apraxia. The extra-ocular motility was otherwise unremarkable. Ocular alignment demonstrated exotropia with no fixation preference and a vertical deviation. The anterior segments of both eyes were within the normal range for her age. Fundus examination of the right eye shows chorioretinal and optic disc colobomas that are affecting fixation; however, the left eye was within normal limits apart from a nasally displaced optic disc. The cyclo-refraction for both eyes was +1.50 diopters. She had a mass that was not present/documented at birth, nor was it seen during the consecutive visits prior to the three-month visit. The mass continued to grow subsequently, with no signs of regression or improvement for two months. The mass had specific characteristics: it was round, red, located at the center of the forehead, and measured 3x2 cm at the time of presentation to the ophthalmology clinic.

Further inspection of the baby showed no other associated skin lesions in the body and no associated lymph node enlargement. Consistent with its clinical and radiological characteristics, the diagnosis of skin capillary hemangioma was made. The family history was unremarkable for any congenital anomalies or metabolic diseases on either side. Both the mother and the father were up-to-date with their vaccinations and following regularly with their primary health care providers. There is a positive history of consanguinity on both sides of the family.

**Figure 3:**
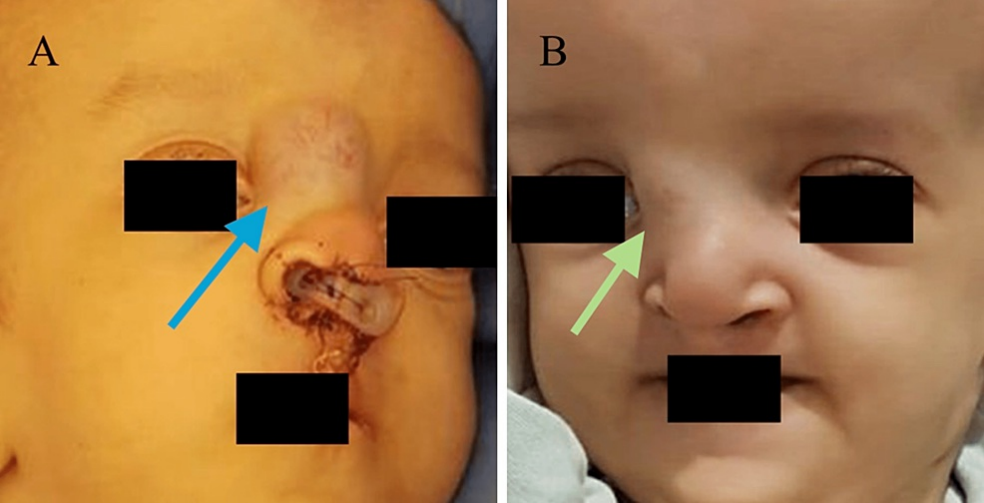
External photo of the face; (A) Capillary hemangioma of the forehead on the first visit (blue arrow). (B) Dramatic reduction in mass size after two months of treatment (green arrow).

Regarding the capillary hemangioma in our patient, the choice to initiate medical treatment was preferred due to its size and location. The patient was started on propranolol syrup 1 mg twice daily. Upon follow-up, the mass decreased in size with no significant complications from the treatment. The dose of propranolol was increased to 2 mg twice daily, for which the patient responded dramatically with a rapid reduction in the mass size, resulting in complete resolution (Figure 3A and Figure 3B).

## Discussion

JS has an unusual and varying clinical picture, with several associated presentations and a myriad of systemic associations observed at the time of diagnosis [2]. A description of the complete associations with JS, moreover, is still a work in progress as the understanding of the diseases and the different contributing genes is still evolving. However, the MTS observed in neuroimaging is a necessary finding essential for diagnosing the disease where it is pathognomonic [2]. Other characteristic findings observed in JS include hypotonia in infancy with a high risk of development of ataxia as the infant grows, developmental delay, intellectual disability, dysmorphic facial features, an abnormal breathing pattern (alternating tachypnea and/or apnea), abnormal eye movements that typically present as oculomotor apraxia or nystagmus [1-3]. Other less frequently encountered findings that occur in less than half of patients with JS include retinal disease, renal disease, coloboma, encephalocele, hepatic disease, and polydactyly [1-3].

Similar to cases in the published literature, the most common ocular findings observed in JS were present in our patient, such as ocular motor apraxia (the most frequent, affecting 80% of reported cases), followed in frequency by strabismus (74%), and nystagmus (72%). A chorioretinal coloboma was also present in our patient, although it was less frequently reported in the literature (30%). The less frequent findings such as ptosis (43% of cases) and optic nerve atrophy (22% of cases) were not present in our patient [1].

Our patient had apnea that was present shortly after birth and facial dysmorphic features in the form of a prominent occiput, wide fontanels, bilateral cleft lip, and a partial cleft palate. Also, present were capillary hemangioma of the forehead, hydrocephalus, hypotonia, and developmental delay. Regarding her ocular findings, the poor fixation pattern is likely related to ocular motor apraxia. It could also be related to the exotropia and the coloboma in her right eye. A cutaneous capillary hemangioma was incidentally found in this JS patient, which responded favorably to medical treatment with propranolol in the form of a significant reduction in the mass's size and, therefore, decreased complications from the mass, such as intra-lesional hemorrhage.

Skin capillary hemangioma in patients with JS is indeed a rare finding. To our knowledge, this is the third reported case in the literature. The value of this report is not so much in the finding itself, but rather, in the potential of the finding being part of the complete picture of JS. Singhi and colleagues in 2007 reported five cases of JS with the typical picture of the disease in all patients apart from one who had a skin capillary hemangioma; they described it as the first reported case in the literature [6]. Later in 2019, Hafeez and colleagues described another case of JS with two atypical findings: first, the presence of Blake’s pouch cyst on radiological imaging, and second, the presence of skin capillary hemangiomas [7]. One further report associates JS with the finding of oral capillary hemangiomas confirmed by histopathology [8].

It is also worth noting that, like our patient, reported cases in the literature with skin [6,7] and oral [8] capillary hemangiomas have a positive family history of consanguinity. This begs the question of whether the genetic element of the disease also gives rise to skin capillary hemangiomas or if they are distinctively separate.

## Conclusions

A cutaneous capillary hemangioma was an incidental finding in this JS patient that responded favorably to medical treatment with propranolol. A significant reduction in mass size was observed preventing local complications from the mass such as intra-lesional hemorrhage. Furthermore, although skin capillary hemangioma is a rare association with JS, it could be part of the complete list of potential associations and warrants further investigation into the presence of a genetic component giving rise to the mass in JS patients or lack thereof.
